# Trends in US Adult Smoking Prevalence, 2011 to 2022

**DOI:** 10.1001/jamahealthforum.2023.4213

**Published:** 2023-12-01

**Authors:** Rafael Meza, Pianpian Cao, Jihyoun Jeon, Kenneth E. Warner, David T. Levy

**Affiliations:** 1 Department of Integrative Oncology, BC Cancer Research Institute, Vancouver, British Columbia, Canada; 2 School of Population and Public Health, University of British Columbia, Vancouver, British Columbia, Canada; 3 Department of Epidemiology, University of Michigan, Ann Arbor; 4 Department of Health Management and Policy, University of Michigan, Ann Arbor; 5 Lombardi Comprehensive Cancer Center, Georgetown University, Washington, DC

## Abstract

**Importance:**

President Biden recently prioritized the fight against smoking as key to reducing cancer mortality.

**Objective:**

To assess trends in smoking and illuminate the association between smoking and reducing deaths due to cancer.

**Design, Setting, and Participants:**

This cross-sectional study used responses to National Health Interview Surveys from January 1, 2011, to December 31, 2022, to characterize trends in current smoking for key sociodemographic groups among US adults.

**Exposures:**

Age (18-24, 25-39, 40-64, and ≥65 years), family income (<200%, 200%-399%, and ≥400% of the federal poverty level [FPL]), educational level (less than high school, high school degree or General Educational Development, some college, and college degree or above), and race and ethnicity (Black, Hispanic, White, and other).

**Main Outcomes and Measures:**

Weighted current smoking prevalence with 95% CIs by analysis group from 2011 to 2022. Average annual percentage change (AAPC) in smoking prevalence by analysis group is calculated using Joinpoint regression.

**Results:**

Data from 353 555 adults surveyed by the National Health Interview Surveys from 2011 to 2022 were included (12.6% Black, 15.0% Hispanic, 65.2% White, and 7.3% other race or ethnicity). Overall, smoking prevalence decreased among adults aged 18 to 24 years from 19.2% (95% CI, 17.5%-20.9%) in 2011 to 4.9% (95% CI, 3.7%-6.0%) in 2022 at an AAPC of −11.3% (95% CI, −13.2% to −9.4%), while it remained roughly constant among adults 65 years or older at 8.7% (95% CI, 7.9%-9.5%) in 2011 and 9.4% (95% CI, 8.7%-10.2%) in 2022 (AAPC, −0.1% [95% CI, −0.8% to 0.7%]). Among adults 65 years or older, smoking prevalence increased from 13.0% (95% CI, 11.2%-14.7%) in 2011 to 15.8% (95% CI, 14.1%-17.6%) for those with income less than 200% FPL (AAPC, 1.1% [95% CI, 0.1%-2.1%]) and remained roughly constant with no significant change for those of higher income. Similar age patterns are seen across educational level and racial and ethnic groups.

**Conclusions and Relevance:**

This cross-sectional study found that smoking prevalence decreased from 2011 to 2022 in all age groups except adults 65 years or older, with faster decreases among younger than older adults. These findings suggest that the greatest gains in terms of reducing smoking-attributable morbidity and mortality could be achieved by focusing on individuals with low socioeconomic status, as this population has the highest smoking rates and the worst health prospects.

## Key Points

**Question:**

Is smoking still decreasing among US adults and do the trends vary by age, income, and race and ethnicity?

**Findings:**

In this cross-sectional study of 353 555 adults responding to the 2011 to 2022 National Health Interview Surveys, adults younger than 40 years had dramatic declines in smoking prevalence during the last decade, especially among those with higher incomes. In contrast, relatively slow declines were observed among adults aged 40 to 64 years, with no decrease in smoking among those 65 years or older.

**Meaning:**

These findings suggest that the precipitous decline in smoking among younger adults should be maintained, but that additional efforts are required to further reduce smoking in older adults.

## Teaser

This cross-sectional study assesses trends from 2011 to 2022 in US adult smoking prevalence by age.

## Introduction

In a White House fact sheet issued prior to President Biden’s 2023 State of the Union speech, the Biden Administration announced that it would “[t]ackl[e] the biggest single driver of cancer deaths in this country—smoking.”^1^ Smoking causes 30% of all cancer deaths and 80% of lung cancer deaths. Peaking in 1990, the age-adjusted male all-cause cancer mortality rate declined by an impressive 38.2% by 2019. Nearly half (47%) of that decrease is attributable to reductions in male lung cancer mortality.^2^ Smoking prevention and treatment will also substantially reduce deaths due to other cancers, chronic obstructive pulmonary disease, heart disease, and stroke. Annually, smoking causes 480 000 US deaths.

Smoking rates can be reduced by continuing the recent rapid declines in smoking initiation by youths and young adults and by increasing cessation among older adults. We characterize trends from 2011 to 2022 in adult smoking prevalence by age, with those at younger ages indicating current patterns of initiation and those at older ages indicating the effects of cessation when smoking-attributable disease and death are most common. We also characterize prevalence trends by income, educational attainment, and race and ethnicity to gauge the association of current initiation and cessation with different socioeconomic groups, particularly those with lower socioeconomic status (SES) who are the most vulnerable due to their high smoking and death rates.

## Methods

This study was approved by the University of Michigan Institutional Review Board and followed the Strengthening the Reporting of Observational Studies in Epidemiology (STROBE) reporting guideline. We applied data from the National Health Interview Surveys (NHISs) from January 1, 2011, to December 31, 2022. For each year, we defined people who currently smoke as those who reported smoking 100 or more cigarettes in their lifetime and smoking every day or some days. Informed consent was not required for the use of deidentified data.

We estimated annual smoking prevalence by age group (18-24, 25-39, 40-64, and ≥65 years) and family income, categorized as a percentage of the survey year’s federal poverty level (FPL).^3^ We used 3 income categories: less than 200% FPL, 200% to 399% FPL, and 400% or greater FPL. We also conducted analyses by race and ethnicity (Black, Hispanic, White, and other race or ethnicity [American Indian or Alaska Native, Asian, other race or ethnicity, or multiple races or ethnicities]) and educational attainment (less than high school, high school degree or General Educational Development, some college, and college degree or above), as smoking prevalence is known to vary considerably by these variables. Analyses by educational level were restricted to those 25 years or older. Sociodemographic data are based on self-responses to the NHIS questionnaires.

Trends in annual weighted smoking prevalence with 95% CIs by analysis group were estimated using SAS, version 9.4 (SAS Institute Inc), accounting for survey sample weights. For each group, we also calculated the average annual percentage change (AAPC) in smoking prevalence from 2011 to 2022 using the National Cancer Institute Joinpoint Regression Program, version 4.9.1.0. The AAPCs are considered significantly different from zero at α = .05.

## Results

**Figure 1.**
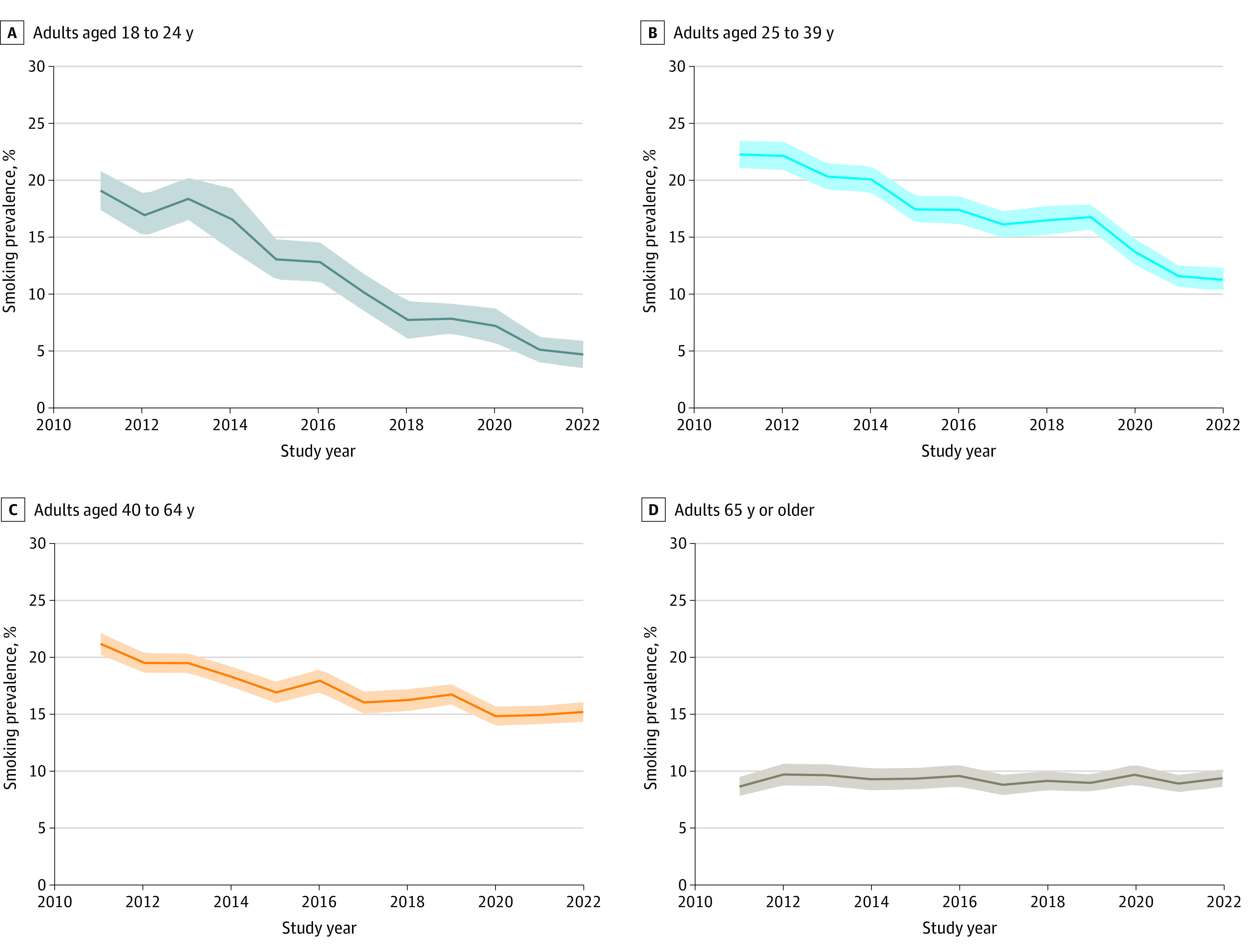
US Annual Smoking Prevalence From 2011 to 2022 by Age Data are from the National Health Interview Surveys. Shading denotes the 95% CI.

Data from 353 555 adults surveyed by the NHIS from 2011 to 2022 were included. Of these, 33.0% had less than 200% FPL income, 29.2% had 200% to 400% FPL income, and 37.8% had greater than 400% FPL income. With respect to race and ethnicity, 12.6% of the sample was Black, 15.0% was Hispanic, 65.2% was White, and 7.3% were of other race or ethnicity. Smoking prevalence decreased from 2011 to 2022 in all age groups except adults 65 years or older, with considerably faster decreases among younger than older adults (Figure 1 and Table). Specifically, smoking prevalence decreased among adults aged 18 to 24 years from 19.2% (95% CI, 17.5%-20.9%) in 2011 to 4.9% (95% CI, 3.7%-6.0%) in 2022 at an AAPC of −11.3% (95% CI, −13.2% to −9.4%); among adults aged 25 to 39 years from 22.4% (95% CI, 21.2%-23.6%) in 2011 to 11.4% (95% CI, 10.5%-12.3%) in 2022 at an AAPC of −5.5% (95% CI, −6.7% to −4.4%); and among adults aged 40 to 64 years from 21.2% (95% CI, 20.3%-22.2%) in 2011 to 15.2% (95% CI, 14.4%-16.1%) in 2022 at an AAPC of −3.0% (95% CI, −3.7% to −2.3%). In contrast, among adults 65 years and older, smoking prevalence increased slightly from 8.7% (95% CI, 7.9%-9.5%) in 2011 to 9.4% (95% CI, 8.7%-10.2%) in 2022 (AAPC, −0.1% [95% CI, −0.8% to 0.7%]).

**Table.** Smoking Prevalence, AAPC From 2011 to 2022 by Age and Family Income

Income group	Smoking prevalence (95% CI), %		AAPC (95% CI), %
	2011	2022	
**Aged 18-24 y**			
All	19.2 (17.5 to 20.9)	4.9 (3.7 to 6.0)	−11.3 (−13.2 to −9.4)^a^
<200% FPL	21.4 (19.0 to 23.8)	6.6 (4.5 to 8.7)	−9.6 (−11.1 to −8.1)^a^
200%-399% FPL	19.8 (16.4 to 23.3)	5.4 (3.3 to 7.6)	−11.0 (−13.4 to −8.5)^a^
≥400% FPL	13.3 (9.4 to 17.3)	2.3 (0.8 to 3.9)	−14.7 (−19.1 to −10.1)^a^
**Aged 25-39 y**			
All	22.4 (21.2 to 23.6)	11.4 (10.5 to 12.3)	−5.5 (−6.7 to −4.4)^a^
<200% FPL	30.5 (28.4 to 32.6)	19.5 (17.2 to 21.7)	−3.7 (−4.9 to −2.5)^a^
200%-399% FPL	22.5 (20.5 to 24.5)	11.1 (9.5 to 12.8)	−5.3 (−6.7 to −3.7)^a^
≥400% FPL	13.7 (12.1 to 15.3)	6.2 (5.1 to 7.2)	−6.8 (−8.3 to −5.2)^a^
**Aged 40-64 y**			
All	21.2 (20.3 to 22.2)	15.2 (14.4 to 16.1)	−3.0 (−3.7 to −2.3)^a^
<200% FPL	32.8 (30.8 to 34.8)	26.1 (24.0 to 28.2)	−2.2 (−2.8 to −1.6)^a^
200%-399% FPL	24.3 (22.6 to 26.0)	17.7 (16.0 to 19.3)	−2.6 (−3.6 to −1.7)^a^
≥400% FPL	13.2 (12.1 to 14.2)	8.8 (7.9 to 9.7)	−3.7 (−4.8 to −2.7)^a^
**Aged ≥65 y**			
All	8.7 (7.9 to 9.5)	9.4 (8.7 to 10.2)	−0.1 (−0.8 to 0.7)
<200% FPL	13.0 (11.2 to 14.7)	15.8 (14.1 to 17.6)	1.1 (0.1 to 2.1)^a^
200%-399% FPL	7.6 (6.4 to 8.9)	9.1 (7.7 to 10.5)	0.8 (−0.9 to 2.6)
≥400% FPL	6.1 (4.7 to 7.5)	5.5 (4.5 to 6.4)	−1.7 (−3.7 to 0.4)

Abbreviation: AAPC, average annual percentage change; FPL, federal poverty level.

^a^ AAPC was statistically significantly different from 0.

**Figure 2.**
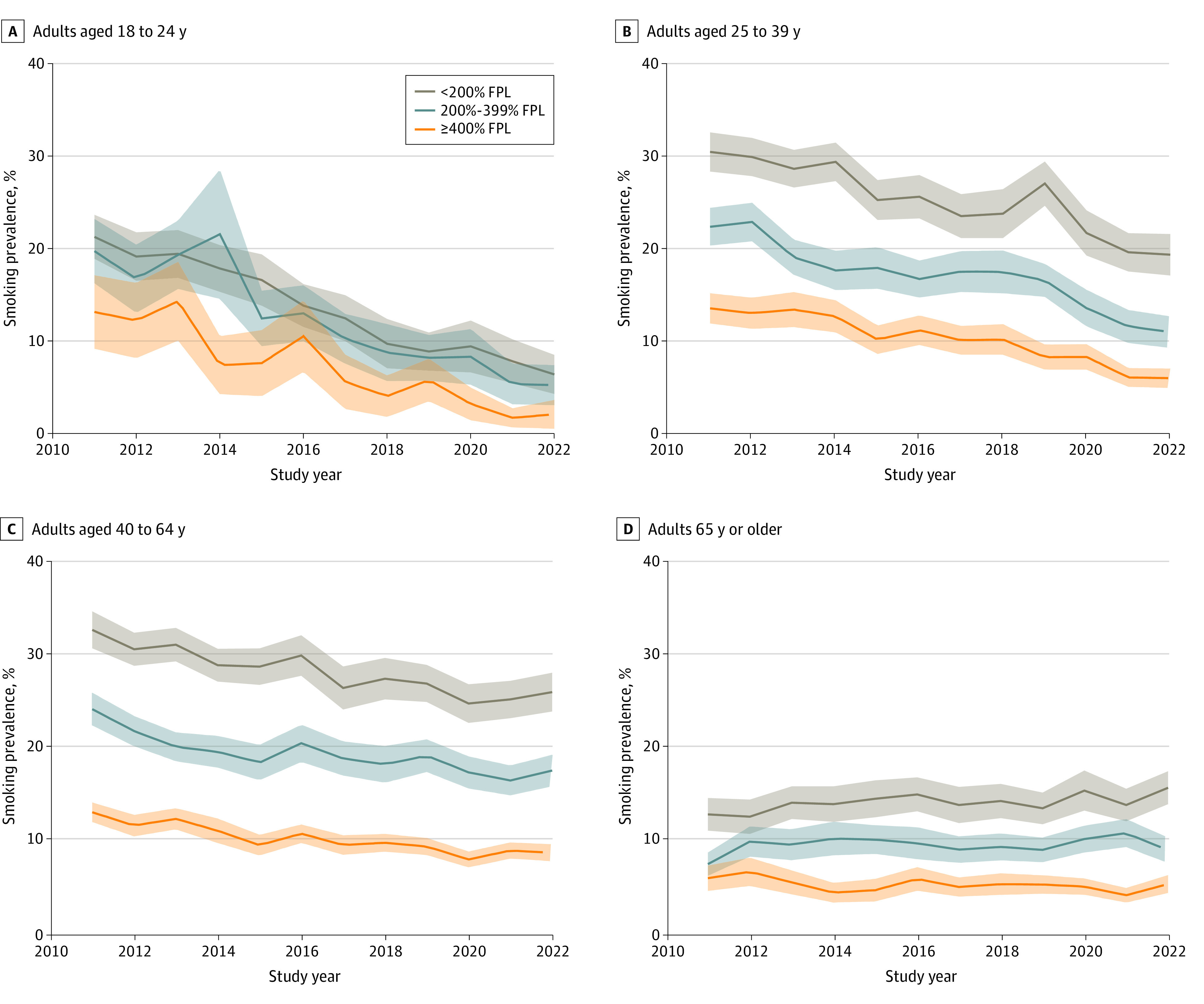
US Annual Smoking Prevalence From 2011 to 2022 by Age and Family Income Data are from the National Health Interview Surveys. Shading denotes the 95% CIs. FPL indicates federal poverty level.

Respondents with the lowest income had the highest smoking prevalence in all age categories. Except for adults 65 years or older (Figure 2 and Table), we observed decreases in prevalence across all income groups, albeit at different rates. Among adults 65 years or older with income less than 200% FPL, smoking prevalence increased from 13.0% (95% CI, 11.2%-14.7%) in 2011 to 15.8% (95% CI, 14.1%-17.6%) in 2022 (AAPC, 1.1% [95% CI, 0.1%-2.1%]) and remained roughly constant with no significant change for those with a higher income. In contrast, among adults aged 18 to 24 years, smoking prevalence decreased for all 3 income levels, albeit with the decreases increasing with income level, at an AAPC of −9.6% (95% CI, −11.1% to −8.1%) for those with income of less than 200% FPL, −11.0% (95% CI, −13.4% to −8.5%) for those with income of 200% to 399% FPL, and −14.7% (95% CI, −19.1% to −10.1%) for those with income of 400% FPL or greater. In every age group, the largest reductions in smoking were among those with higher income.

Trends by educational level and race and ethnicity are presented in eTables 1 and 2 in Supplement 1. Similar age patterns are observed with considerable decline in smoking prevalence across all educational levels and race and ethnicity groups at younger ages and relatively constant or nondecreasing trends at older ages (eFigures 1 to 3 in Supplement 1). By educational level, among adults 65 years or older, smoking prevalence significantly decreased only among those with educational attainment of college or above, with a significant increase among those with educational attainment of high school or GED. By race and ethnicity, among adults 65 years or older, smoking prevalence remained constant across all groups, with non–statistically significant increases among Black, Hispanic, and other racial and ethnic groups.

## Discussion

While overall US adult smoking prevalence continues to decline, the constant or increasing trends among those 65 years or older and the relatively slow decreases among adults aged 40 to 64 years, especially for those with lower income and educational levels, are striking. In contrast, those younger than 40 years, particularly those aged 18 to 24 years, have seen dramatic declines in a relatively short period, reaching historically low levels of smoking prevalence, especially among those with higher incomes. While smoking trends since 2019 may reflect the impact of COVID-19, no clear patterns have emerged.

Recent studies have also reported low and rapidly declining rates of smoking among youths.^4,5^ Our findings indicate that the decreases in youth initiation of smoking are now translating into rapid declines in smoking prevalence among young adults. This suggests further declines in adult prevalence as newer generations with low smoking levels continue to age. However, while the future looks promising for younger populations, relatively constant trends in smoking prevalence among adults 65 years or older and the relatively slow declines among adults aged 40 to 64 years are concerning, since most smoking-related deaths occur at older ages. Increasing smoking cessation, particularly among middle-aged and older adults, is thus critical to further reduce smoking-attributable mortality. Our results underscore the importance of responding to recent calls for increasing support for smoking cessation programs by the US Department of Health and Human Services,^6^ the US Food and Drug Administration,^7,8^ and the US Preventive Services Task Force.^9^

The lack of decline among those with low SES (low income or low educational level) and racial and ethnic minority individuals 65 years or older and the consistently higher smoking prevalence among individuals with lower incomes in all age groups indicate the need to target cessation efforts to reduce disparities.^10^ A ban on menthol in cigarettes and cigars is associated with reduced racial disparities.^11^ Increasing taxes on combustible tobacco products in low-taxing states may reduce disparities across states.^12^ These targeted interventions would reduce health disparities and improve health equity by reducing smoking-attributable deaths among those with lower SES.^10^

The use of e-cigarettes may also play a role.^13^ Increases in e-cigarette use have accompanied substantial reductions in smoking by youths and young adults, although a causal relationship has not been established.^4,5^ While e-cigarette use is currently much less common among older adults,^14^ e-cigarettes could play an important role as a smoking cessation aid for older adults.^15^

### Limitations

This study has some limitations. First, we did not consider other tobacco products, such as cigars or e-cigarettes. Second, due to sample size limitations, we did not assess trends at the intersection of income or education and race and ethnicity. Third, we did not consider youth tobacco use.

## Conclusions

This cross-sectional study found that smoking prevalence decreased from 2011 to 2022 among all age groups except adults 65 years or older, with faster decreases among younger than older adults. These findings suggest that the greatest gains in reducing smoking-attributable mortality could be attained by focusing efforts on smokers with low SES, the group with the highest smoking rates and worst health prospects.

## Notes

Supplement 1. **eTable 1.** Smoking Prevalence Average Annual Percentage Change (AAPC) From 2011 to 2022 by Age and Educational Attainment **eTable 2.** Smoking Prevalence Average Annual Percentage Change (AAPC) From 2011 to 2022 by Age and Race and Ethnicity **eFigure 1.** US Annual Smoking Prevalence From 2011 to 2022 by Age and Educational Attainment (National Health Interview Survey) **eFigure 2.** US Annual Smoking Prevalence From 2011 to 2022 by Age and Race and Ethnicity (National Health Interview Survey) **eFigure 3.** US Annual Smoking Prevalence From 2011 to 2022 by Age and Race and Ethnicity With 95% CIs (National Health Interview Survey)

Supplement 2.
Data Sharing Statement
