# Supplementary material for: Trends in US Adult Smoking Prevalence, 2011 to 2022
Source: JAMA Health Forum. 2023 Dec 1;4(12):e234213. doi: 10.1001/jamahealthforum.2023.4213 (PMC10692849; doi:10.1001/jamahealthforum.2023.4213)
Supplement: Supplement 1. — eTable 1. Smoking Prevalence Average Annual Percentage Change (AAPC) From 2011 to 2022 by Age and Educational Attainment eTable 2. Smoking Prevalence Average Annual Percentage Change (AAPC) From 2011 to 2022 by Age and Race and Ethnicity eFigure 1. US Annual Smoking Prevalence From 2011 to 2022 by Age and Educational Attainment (National Health Interview Survey) eFigure 2. US Annual Smoking Prevalence From 2011 to 2022 by Age and Race and Ethnicity (National Health Interview Survey) eFigure 3. US Annual Smoking Prevalence From 2011 to 2022 by Age and Race and Ethnicity With 95% CIs (National Health Interview Survey) [file jamahealthforum-e234213-s001.pdf]

# Supplementary Online Content

Meza R, Cao P, Jeon J, Warner KE, Levy DT. Trends in US adult smoking prevalence, 2011 to 2022. *JAMA Health Forum*. Published online December 1, 2023. doi:10.1001/jamahealthforum.2023.4213

**eTable 1.** Smoking Prevalence Average Annual Percentage Change (AAPC) From 2011 to 2022 by Age and Educational Attainment
**eTable 2.** Smoking Prevalence Average Annual Percentage Change (AAPC) From 2011 to 2022 by Age and Race and Ethnicity
**eFigure 1.** US Annual Smoking Prevalence From 2011 to 2022 by Age and Educational Attainment (National Health Interview Survey)
**eFigure 2.** US Annual Smoking Prevalence From 2011 to 2022 by Age and Race and Ethnicity (National Health Interview Survey)
**eFigure 3.** US Annual Smoking Prevalence From 2011 to 2022 by Age and Race and Ethnicity With 95% CIs (National Health Interview Survey)

This supplementary material has been provided by the authors to give readers additional information about their work.

© 2023 Meza R et al. *JAMA Health Forum*.

eTable 1. Smoking Prevalence Average Annual Percentage Change (AAPC) from 2011 to 2022 by Age and Educational Attainment

| Age Group | Education Group | 2011 Smoking Prevalence (percent; 95% CI) | 2022 Smoking Prevalence (percent; 95% CI) | AAPC (percent; 95% CI) |
|---|---|---|---|---|
| **25-39** | Less than HS | 30.4 (26.7, 34.1) | 22.0 (17.6, 26.4) | **-2.1* (-3.4, -0.7)** |
| **25-39** | HS or GED | 33.3 (30.6, 36.0) | 19.1 (16.7, 21.5) | **-4.9* (-5.9, -3.9)** |
| **25-39** | Some College | 26.0 (24.0, 28.0) | 12.6 (10.8, 14.4) | **-5.0* (-6.3, -3.6)** |
| **25-39** | College or above | 8.7 (7.5, 9.9) | 3.7 (3.0, 4.5) | **-7.9* (-9.6, -6.2)** |
| | | | | |
| **40-64** | Less than HS | 32.8 (29.9, 35.7) | 26.9 (23.8, 30.0) | **-2.2* (-3.1, -1.3)** |
| **40-64** | HS or GED | 31.0 (29.1, 32.9) | 22.4 (20.5, 24.4) | **-2.8* (-3.6, -1.9)** |
| **40-64** | Some College | 22.0 (20.4, 23.6) | 16.5 (15.0, 18.1) | **-2.7* (-3.6, -1.7)** |
| **40-64** | College or above | 7.8 (6.8, 8.8) | 5.4 (4.6, 6.2) | **-3.7* (-5.3, -2.0)** |
| | | | | |
| **65+** | Less than HS | 12.0 (9.7, 14.3) | 17.4 (14.2, 20.7) | 1.8 (-0.6, 4.4) |
| **65+** | HS or GED | 9.6 (8.2, 11.0) | 11.4 (9.8, 12.9) | **1.2* (0.1, 2.3)** |
| **65+** | Some College | 9.0 (7.2, 10.8) | 9.3 (8.1, 10.6) | -0.4 (-2.1, 1.3) |
| **65+** | College or above | 4.9 (3.6, 6.3) | 4.3 (3.4, 5.2) | **-2.1* (-3.6, -0.6)** |

* - AAPC statistically significantly different from 0.

CI- Confidence interval; HS – High school; GED – General Equivalence Degree.

© 2023 Meza R et al. *JAMA Health Forum*.

eTable 2. Smoking Prevalence Average Annual Percentage Change (AAPC) from 2011 to 2022 by Age and Race/Ethnicity

| Age Group | Race/Ethnicity Group | 2011 Smoking Prevalence (percent; 95% CI) | 2022 Smoking Prevalence (percent; 95% CI) | AAPC (percent; 95% CI) |
|---|---|---|---|---|
| **18-24** | NHW | 23.2 (20.7, 25.7) | 6.4 (4.4, 8.3) | **-11.4* (-13.5, -9.1)** |
| **18-24** | NHB | 14.6 (11.3, 17.9) | 2.0 (0.4, 3.5) | **-11.7* (-15.9, -7.4)** |
| **18-24** | Hispanics | 11.6 (9.1, 14.1) | 3.1 (1.6, 4.6) | **-11.2* (-14.4, -8.0)** |
| **18-24** | NH Other | 16.0 (10.6, 21.4) | 4.6 (1.7, 7.5) | **-6.5* (-10.4, -2.4)** |
| | | | | |
| **25-39** | NHW | 26.6 (24.9, 28.3) | 12.5 (11.1, 13.9) | **-5.9* (-7.1, -4.7)** |
| **25-39** | NHB | 21.3 (18.7, 23.9) | 14.8 (11.6, 17.9) | **-4.0* (-5.7, -2.3)** |
| **25-39** | Hispanics | 13.5 (11.6, 15.3) | 8.3 (6.7, 9.9) | **-4.5* (-5.9, -3.0)** |
| **25-39** | NH Other | 13.9 (11.2, 16.6) | 7.8 (5.7, 10.0) | **-3.7* (-6.7, -0.6)** |
| | | | | |
| **40-64** | NHW | 22.7 (21.5, 23.8) | 17.1 (16.0, 18.2) | **-2.5* (-3.3, -1.8)** |
| **40-64** | NHB | 22.6 (20.5, 24.7) | 18.0 (15.4, 20.5) | **-2.8* (-3.7, -1.9)** |
| **40-64** | Hispanics | 14.2 (12.3, 16.1) | 9.6 (8.0, 11.3) | **-3.9* (-5.3, -2.4)** |
| **40-64** | NH Other | 15.1 (12.1, 18.1) | 9.2 (7.0, 11.5) | **-2.7* (-5.2, -0.1)** |
| | | | | |
| **65+** | NHW | 8.6 (7.7, 9.6) | 9.1 (8.2, 9.9) | -0.2 (-1.0, 0.5) |
| **65+** | NHB | 12.0 (9.4, 14.6) | 14.9 (11.9, 17.8) | 0.8 (-2.0, 3.8) |
| **65+** | Hispanics | 8.0 (5.8, 10.2) | 9.0 (6.2, 11.7) | 0.1 (-2.9, 3.2) |
| **65+** | NH Other | 5.4 (3.6, 7.1) | 6.3 (3.6, 9.0) | 3.0 (-0.8, 6.9) |

* - AAPC statistically significantly different from 0.

CI – Confidence Interval; NHW – non-Hispanic Whites; NHB – non-Hispanic Blacks; NH Other - non-Hispanic Others.

© 2023 Meza R et al. *JAMA Health Forum*.

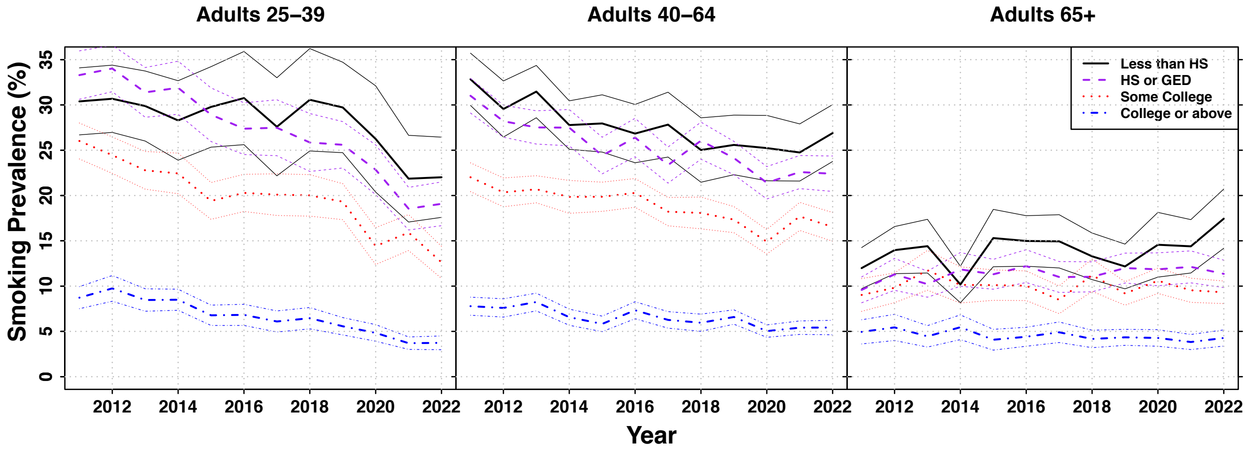


eFigure 1. US annual smoking prevalence from 2011 to 2022 by age and educational attainment (National Health Interview Survey). HS – High school; GED- General Equivalence Degree. Thinner lines denote 95% confidence intervals.

© 2023 Meza R et al. *JAMA Health Forum*.

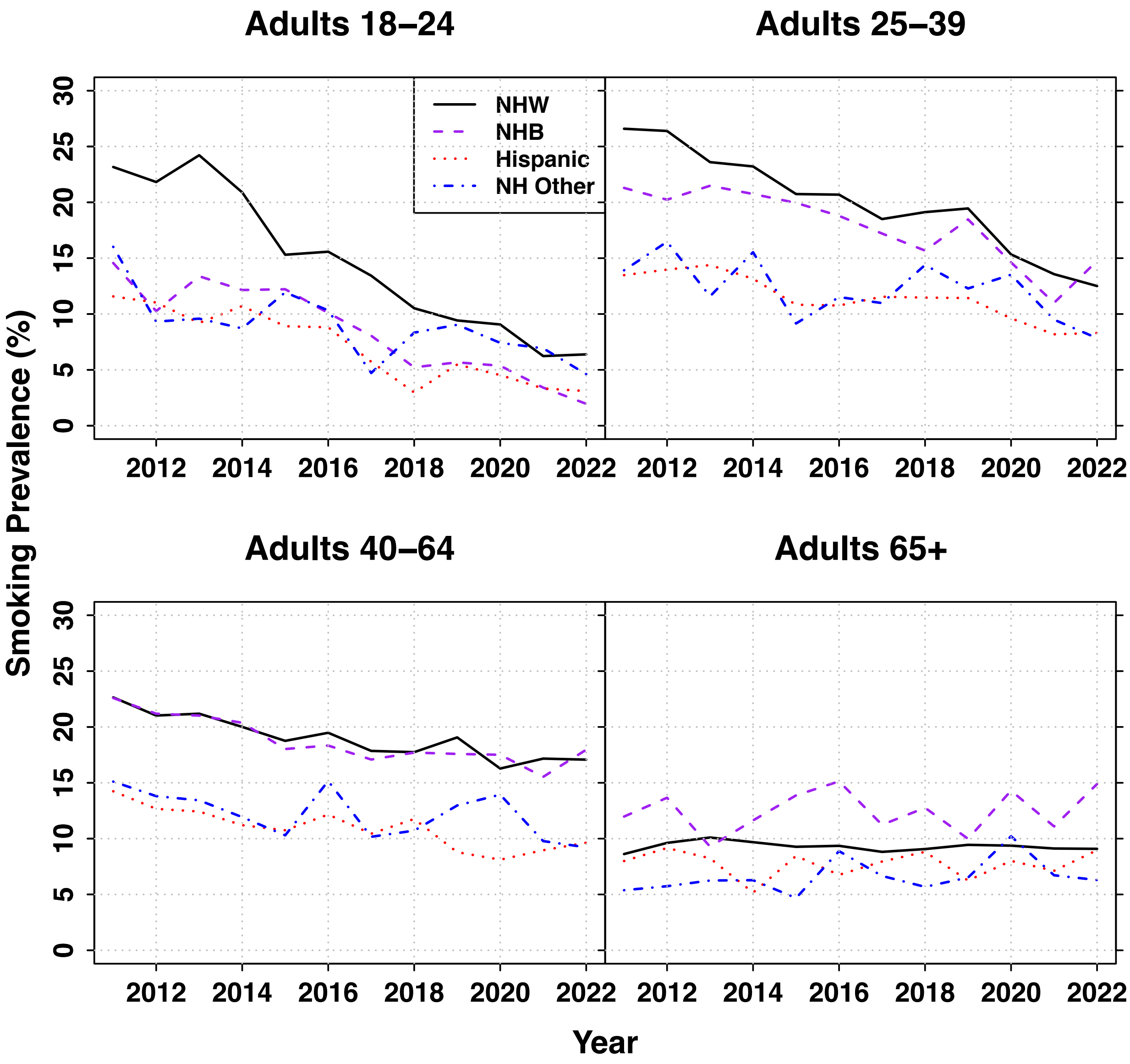


eFigure 2. US annual smoking prevalence from 2011 to 2022 by age and race/ethnicity (National Health Interview Survey). NHW – non-Hispanic Whites; NHB – non-Hispanic Blacks; NH Other - non-Hispanic Others. Corresponding 95% confidence intervals are shown in Figure S3.

© 2023 Meza R et al. *JAMA Health Forum*.

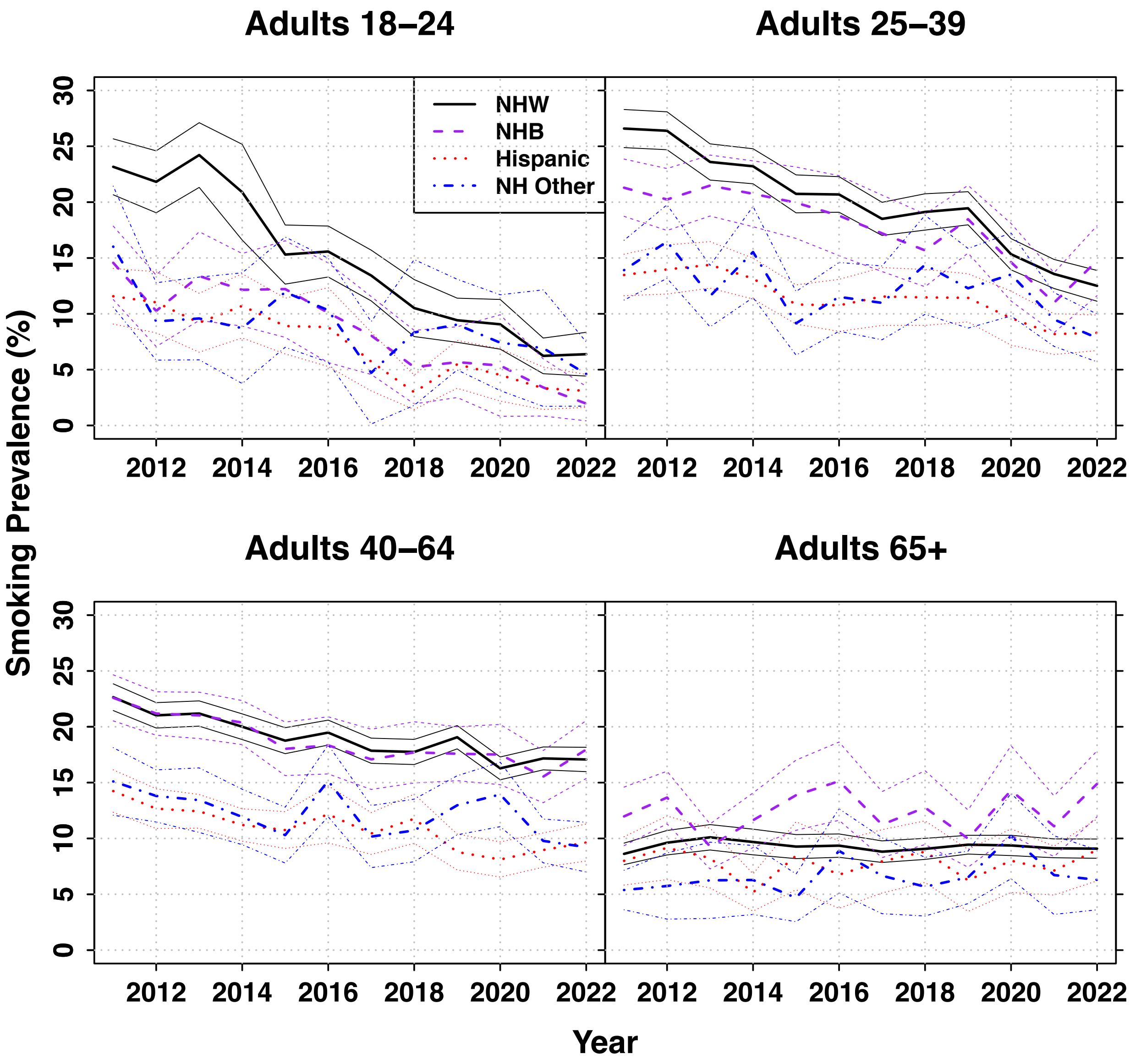


eFigure 3. US annual smoking prevalence from 2011 to 2022 by age and race/ethnicity (National Health Interview Survey). NHW – non-Hispanic Whites; NHB – non-Hispanic Blacks; NH Other - non-Hispanic Others. Thinner lines denote 95% confidence intervals.

© 2023 Meza R et al. *JAMA Health Forum*.
