# Supplementary material for: Trends in US Adult Smoking Prevalence, 2011 to 2022
Source: JAMA Health Forum. 2023 Dec 1;4(12):e234213. doi: 10.1001/jamahealthforum.2023.4213 (PMC10692849; doi:10.1001/jamahealthforum.2023.4213)
Supplement: Supplement 2. — Data Sharing Statement [file jamahealthforum-e234213-s002.pdf]

Data Sharing Statement

Meza. Trends in US Adult Smoking Prevalence, 2011 to 2022. *JAMA Health Forum*. Published December 01, 2023. doi:10.1001/jamahealthforum.2023.4213

Data
Data available: No

Additional Information
Explanation for why data not available: NHIS data is publicly available
